# Reverse Phase Protein Arrays—Quantitative Assessment of Multiple Biomarkers in Biopsies for Clinical Use

**DOI:** 10.3390/microarrays4020098

**Published:** 2015-03-24

**Authors:** Stefanie Boellner, Karl-Friedrich Becker

**Affiliations:** Institut für Pathologie, Technische Universität München, Trogerstrasse 18, 81675 München, Germany; E-Mail: stefanie.boellner@tum.de

**Keywords:** tissue, cancer, personalized medicine, FFPE, protein, antibody, diagnostics, therapy

## Abstract

Reverse Phase Protein Arrays (RPPA) represent a very promising sensitive and precise high-throughput technology for the quantitative measurement of hundreds of signaling proteins in biological and clinical samples. This array format allows quantification of one protein or phosphoprotein in multiple samples under the same experimental conditions at the same time. Moreover, it is suited for signal transduction profiling of small numbers of cultured cells or cells isolated from human biopsies, including formalin fixed and paraffin embedded (FFPE) tissues. Owing to the much easier sample preparation, as compared to mass spectrometry based technologies, and the extraordinary sensitivity for the detection of low-abundance signaling proteins over a large linear range, RPPA have the potential for characterization of deregulated interconnecting protein pathways and networks in limited amounts of sample material in clinical routine settings. Current aspects of RPPA technology, including dilution curves, spotting, controls, signal detection, antibody validation, and calculation of protein levels are addressed.

## 1. Introduction

The aim of future medicine is personalized treatment which is often characterized as providing the right patient with the right drug at the right dose at the right time. An increasing number of targeted adjuvant cancer therapies, *i.e.*, any treatment given after primary therapy (e.g., surgery), is already available. An example for the success of such novel treatment strategies is the monoclonal antibody trastuzumab against the receptor tyrosine kinase HER2 [1]. Breast cancer and gastric cancer patients who will most likely respond to this therapy are identified before treatment by molecular tests, such as immunohistochemistry (IHC) and fluorescence *in situ* hybridization (FISH). In the case of a neoadjuvant setting, *i.e.*, a treatment prior to surgery, patients who most likely will benefit from a treatment have to be identified using limited amounts of tissues, most often small biopsies with varying percentages of tumor cells. A tumor-specific treatment before surgery can increase the chance of tumor-free resection and therefore increase the overall survival [2]. As tiny biopsies are the most common available tumor samples in the neoadjuvant settings and on the other hand the number of predictive biomarkers for treatment decisions is increasing, highly multiplex methods for quantitative tumor analysis with low sample consumption are needed. 

While human cancers evolve from benign to malignant lesions by acquiring a series of gene mutations over time [3], the results of gene mutations translate to dysregulation or dysfunction of proteins, including kinases, which are attractive drug targets [4]. Currently, many cancer therapeutics are designed to target the malfunction of intracellular signaling pathways that rely on de- or phosphorylated proteins. Thus, to apply personalized medicine more efficiently it is necessary to analyze tumors at the protein level in addition to determining gene mutations or gene expression patterns. 

There are several methods available for protein analysis. Each of them has advantages and disadvantages that are listed in Table 1. For example, Western Blot and Enzyme Linked Immunosorbent Assay (ELISA) require high amounts of protein lysates. Therefore, we think that despite their advantages (e.g., protein separation according to molecular weight and easy quantification, respectively) the use of Western Blot and ELISA is not reasonable in clinical routine for detection of deregulated signaling pathways when only biopsies are available for the analysis. Currently, for protein analysis of tumor tissues IHC is mainly used which gives a spatial resolution of the epitopes analyzed down to a single cell. Using IHC and the tissue microarray (TMA) technology, it is now possible to assay hundreds of patient tissues arrayed on a single microscope slide [5]. However, IHC can hardly provide information about the activation status of proteins as the detection limit of the method is often not sufficient for analyzing phosphorylated proteins. The importance of detecting phosphoproteins in tumor samples is illustrated by a recent study that revealed that patients with HER2 negative breast cancers (IHC/FISH) express a phosphorylated form of HER2 [6]. Thus, these phospho‑HER2 positive patients may benefit from anti-HER2 treatments in addition to patients showing HER2 gene amplification. Mass spectrometry (MS)-based technologies have rapidly advanced in recent years. Beside other advantages mentioned in Table 1, MS-based methods enable the identification of new biomarker candidates by comparing protein signals obtained from cancer and healthy tissues (*de novo* discovery platform) [7,8,9,10]. However, in our opinion, MS‑based technologies are not suitable for the use in clinical routine at the moment. First, due to the complex sample preparation and secondly due to the insufficient profiling of low‑abundance signaling proteins.

**Table 1:** Advantages and disadvantages of commonly used protein analysis platforms for tissue samples.

Protein analysis platform	Advantages	Disadvantages
Western Blot	Separation of proteins according to molecular weight	Work-intensive, high amounts of protein lysate required, low- or medium-throughput
ELISA	Quantitative, very sensitive	High amounts of protein lysate required
IHC	Cellular localization of protein of interest	Semi-quantitative, sensitivity often not sufficient to detect phosphorylated proteins
Mass spectrometry-based technologies	*De novo* discovery platform, highly multiplex, protein isoforms can be distinguished, analysis of thousands of proteins, no protein binding reagent required	Complex sample preparation, poor analytical sensitivity compared to immunoassays, low-throughput
Forward Phase Protein Arrays	Many analytes can be measured in parallel in a single sample, quantitative	Two highly specific antibodies are needed for every assay, high amounts of protein lysate required
Reverse Phase Protein Arrays	Robust quantification, low amount consumption, high-throughput, highly sensitive, detection of phosphoproteins possible	One highly specific antibody is needed for every assay, special devices needed

Abbreviations: IHC: Immunohistochemistry, ELISA: Enzyme Linked Immunosorbent Assay.

**Figure 1:**
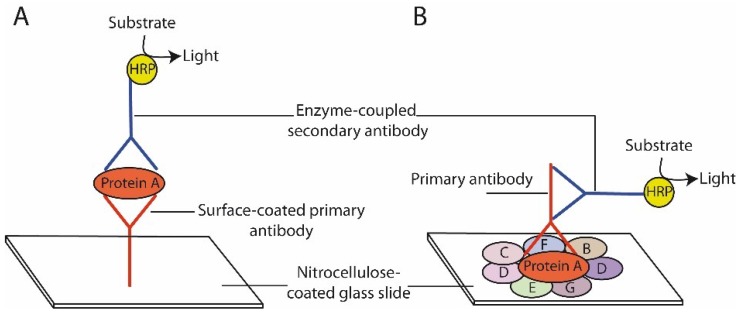
Schematic presentation of forward phase protein array (**A**) and reverse phase protein array (**B**). Protein A: Protein of interest. B–G: Proteins of the lysate that are not recognized by the primary antibody. HRP: Horse radish peroxidase.

Different kinds of very sensitive protein arrays have been developed to quantitatively measure protein levels in high-throughput and multiplex formats. Most of them either function on the basis of forward phase protein arrays or reverse phase protein arrays [11]. In the case of forward phase protein arrays numerous capture antibodies are printed on a solid phase and are exposed to a single protein lysate (Figure 1), allowing detection of multiple proteins in a single sample. The disadvantage is, however, that two highly specific antibodies are needed for protein detection and considerable amounts of starting material are required.

The technology of Reverse Phase Protein Arrays (RPPA), a miniaturized dot blot, was first described in 2001 and is based on the simultaneous measurement of a single protein in multiple samples (Figure 1) [12]. RPPA has proven to be useful for efficient tissue protein quantification and for analysis of signaling cascades even on the basis of very low amounts of tumor sample (biopsy), enabling quantification of biomarkers in tumors in neoadjuvant settings and at early time points [13,14,15]. Using RPPA even phosphoproteins can be detected. Thus, RPPA represents a very powerful tool to identify patients that most likely will benefit from future targeted therapies, aiming to inhibit deregulated kinases. The power of RPPA is reflected by an increasing number of groups worldwide that study protein levels in various sample material including human and animal tissues and cell lines [16,17,18,19,20,21]. 

We will now highlight the clinical use of RPPA and then discuss in detail recent developments for optimizing the RPPA procedure.

## 2. Clinical Applications of RPPA

RPPA is increasingly being used to determine deregulated signaling networks in cancer tissues. For example, RPPA was one of the methods to determine if multi-omic molecular profiling based treatment improves the clinical course of patients with metastatic breast cancer measured by growth of modulation index (GMI). GMI was calculated as the ratio of length of time between treatment and further growth of the primary tumor or metastases. Multi-omic molecular profiling-based therapy differed in all 25 patients from the treatment selected by the treatment selection committee. It could be shown that the molecular profiling-rationalized treatment recommendation can improve the progression free time period, indicating RPPA as a suitable tool to select an appropriate anti-cancer therapy [22]. Besides profiling signaling pathways or entire networks in human cancer tissues, one of the three most common methods to validate mass spectrometry discovered biomarkers is RPPA [23]. If a validated antibody is available for a potential new biomarker, RPPA can be used to validate independent patient-derived sample sets, separate from those initially used for discovery [24].

RPPA analyses have not only focused on protein detection for biomarker discovery and quantification but also to analyze protein expression profile changes during the pre-analytical phase. In several publications, it has been shown that tissue is still alive after resection and gene expression as well as protein levels (especially amounts of phosphoproteins) can change during cold ischemia [25,26,27], *i.e.*, the time period from resection until sample stabilization. One of the more recent publications investigated multiple tissue samples from the same specimen in different patients using targeted and non-targeted technologies [28]. Besides mass spectrometry, RPPA was used to determine potential fluctuations of protein levels. The data allowed the classification of protein and phosphoprotein levels during the pre-analytical phase in three groups: (1) predictable stable; (2) predictable unstable; (3) unpredictable. As most phosphoproteins belonged to the latter group, the authors recommend tissue fixation or stabilization after specimen collection without delay. Thus, tissue procurement guidelines should be aligned [29]. 

RPPA has also been used to analyze heterogeneity of protein levels within a tumor and in primary tumor and lymph node metastases of the same patient [30,31,32,33]. All of these studies revealed a significant heterogeneity of a subset of proteins within a tumor and between primary tumor and metastases, suggesting that for molecular diagnosis the use of multiple tumor samples from distinct locations rather than the analysis of one single sample should be envisaged.

## 3. Reverse Phase Protein Array-Procedure

**Figure 2:**
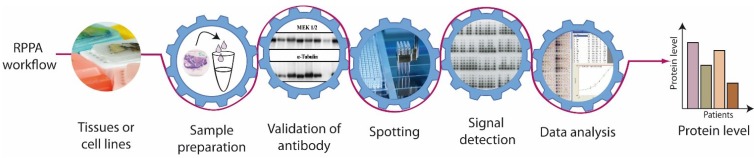
Workflow of Reverse Phase Protein Array (RPPA) studies.

For application in clinical routine RPPA needs to be reproducible and easy to handle to allow high‑throughput testing. Key steps in the establishment of a reliable protein microarray-based procedure are (i) sample preparation, (ii) antibody validation, (iii) spotting, (iv) signal detection, and (v) data analysis (Figure 2). 

### 3.1. Sample Preparation

Tissue samples that are suitable for RPPA can be fresh, frozen, or fixed. So far it is a world-wide standard to use formalin-fixation and paraffin-embedded (FFPE) tissue samples for histopathological diagnosis. Huge archives of patient samples are available in the Institutes of Pathology. The advantage of FFPE-tissues compared to frozen tissue samples are multiple: (i) routine method of all Institutes of Pathology worldwide, (ii) broadly available, (iii) linkage of molecular data with clinical information, (iv) tissues of rare diseases are available, (v) cheap and (vi) easy storage of tissue blocks for many years or even decades at room temperature [34]. The problem of formalin-fixed tissues is that extensive cross-linking of macromolecules hinders the easy extraction of proteins. With the introduction of antigen retrieval techniques in the early 1990s in the pathology laboratories [35], IHC has become manifest as the only routinely used technique to analyze proteins in FFPE-tissue. This may change in the future due to the development of protocols allowing the extraction of full-length proteins from FFPE-tissues. Thus, extraction-based protein analysis is likely to complement IHC data. Histologic evaluation of the samples by a pathologist before protein extraction is needed to estimate the cellular composition. Often it is desired to analyze a specific subpopulation of cells in a tissue section, e.g., tumor cells. Tissue microdissection for cell enrichment can be performed either laser‑assisted or manually. The purification of pure tumor cell populations by laser-capture microdissection (LCM) has been efficiently coupled to RPPA for studying tumor biopsies and detection of clinically relevant information on the molecular characteristics of tumor cells [36]. The so‑called “guided protein extraction” using manual tissue microdissection is an alternative technology [37]. So far, several groups have reported different protocols for extractions of proteins from FFPE-tissues [34,38,39,40,41,42,43]. We recommend deparaffinization (e.g., with xylol) and rehydration with a graded series of alcohol (see also [38]). For protein extraction a heating step is crucial in all mentioned protocols. Additionally, Chu *et al.* propose an optional implementation of microwave or ultrasound to improve the reversal of protein-crosslinking and the penetration of buffer into the tissue [39]. Chung *et al.* use a combination of heat and pressure to obtain optimal protein extraction [40]. Most buffers used for protein extraction usually include detergents (e.g., 1%–2% SDS) for optimal solubilization of hydrophobic proteins. In our lab, we use Qproteome FFPE-Tissue Kit-Buffer (Qiagen, Hilden, Germany), because it is compatible with our antibodies and allows high quality protein extraction [34]. Furthermore, it does not interfere with Sypro Ruby staining which is necessary for measuring the amount of total protein printed on nitrocellulose-coated glass slides. It should be emphasized that different applications may require different extraction conditions and that samples or buffers with high viscosity are not suitable for certain printing platforms. With our protein extraction method, it could be shown that the HER2 score of breast cancer samples obtained by RPPA is of good concordance with the HER2 score obtained with IHC [44]. Beyond that, it could be demonstrated that data obtained from RPPA of cryo‑frozen samples were in agreement with data of RPPA analyzed FFPE samples, indicating that FFPE-tissues are a valuable source for protein level analysis by RPPA [45]. Although most investigators accept that proteins extracted from FFPE tissue are suitable for downstream proteomic analysis, including RPPA [46], the workflows for collecting specimens are not standardized between hospitals, or even within single institutions. Thus, the pre-analytical phase must be improved in order to develop more reliable biomarkers and more effective treatments. There are major efforts to meet the needs as the European Committee for Standardization (CEN) will publish Technical Specifications in 2015, aiming to reduce errors in the pre-analytical phase [47]. There are indications that these European Standards will proceed to global Standards on the ISO (International Organization for Standardization) level. In addition to fresh, frozen or FFPE-tissue samples, types of samples that have already been used for RPPA analysis include cellular lysates obtained by laser capture microdissection [36], serum [48,49], body fluids [50], cell culture lysates [17,51], low molecular weight serum protein fractions [52], peptides [53] and fine needle aspirates [54,55], which reflects the sample diversity for which RPPA can be used.

### 3.2. Antibody Validation

**Figure 3:**
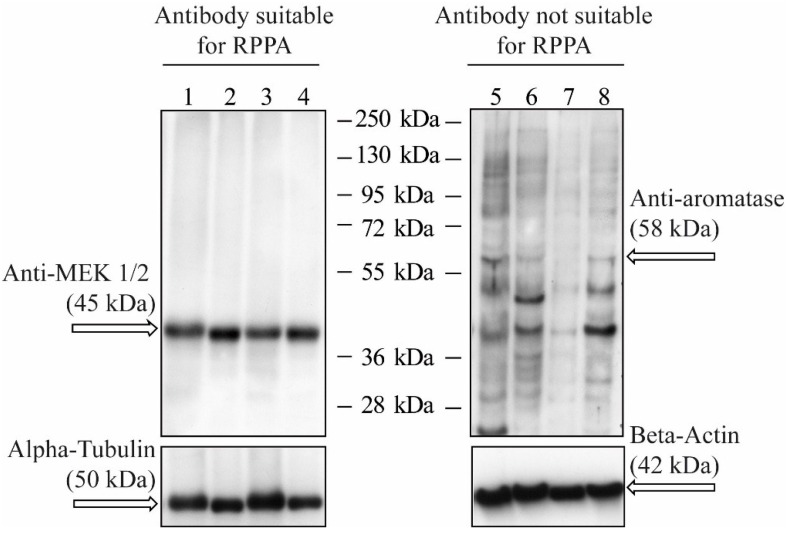
Antibody validation by Western Blot using different cell lines. The left panel shows an antibody that is suitable for RPPA analysis as it detects one single protein band at the expected molecular weight. The antibody used for the right panel should not be used for RPPA studies as there are numerous bands besides the proposed “specific” band. 1–4: lysates from breast cancer FFPE-tissue; 5–8: lysates from different cell lines; arrows indicate the expected molecular weight.

**Figure 4:**
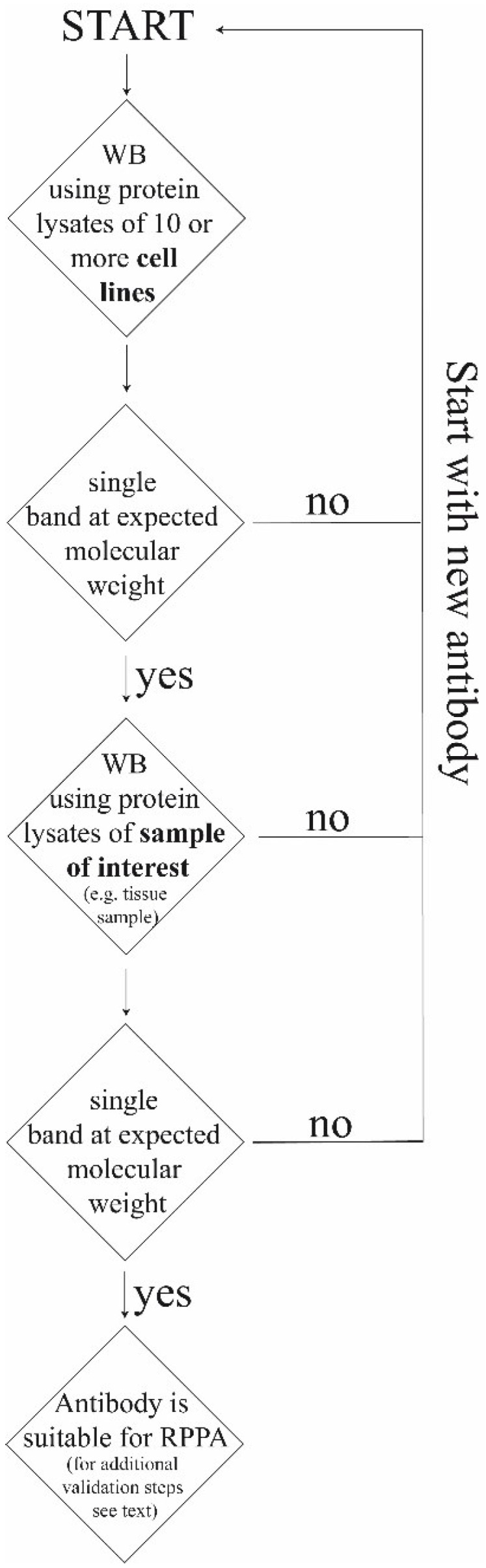
Basic steps for antibody validation. For detailed explanations of the different steps see text. WB, Western Blot.

Reliability of RPPA results largely depends on the quality of the antibodies as signals cannot be differentiated in “specific” and “unspecific” like for conventional immunoblotting techniques. Unfortunately, commercial available antibodies often do not meet the demand necessary for RPPA. For antibody validation Schuster *et al.* propose a step-by-step procedure combining immunohistochemistry and immunoblot analysis from FFPE-tissues using the same antibody. This kind of tandem validation identifies antibodies that bind specific to the respective antigen at the right molecular weight (Western Blot) and allows identification of antibodies that can localize the protein of interest in tissue sections (IHC). By combining Western Blot and IHC antibody validation could be improved [56]. Alternatively, protein extracts of several different cell lines (ten or more) can be a starting point for validation. The first criterion for a specific antibody is a single band at the correct molecular weight for protein extracts of cell lines obtained by Western Blot (Figure 3). The antibody may also be considered specific, if several protein bands appear that correspond to isoforms, cleavage products, dimer formation or mutations (*i.e.*, “explainable bands” as for example for ERK1/2 or p95 and p185 in the case of HER2). Often, antibodies are tested against stimulated and unstimulated cell lines in order to detect phosphorylated proteins [57,58,59]. After analysis of cell lysates, the next step for antibody validation is Western Blot analysis using protein lysates of the material which will be used for RPPA later on, e.g., frozen or FFPE‑tissue. This step is crucial because antigen detection in cell lines might deviate from results obtained in tissues samples [56]. In a further test, quantification results obtained by RPPA are compared with those of Western Blot. If the results are comparable, e.g., stronger staining in IHC and higher protein amounts in the same cases by RPPA analysis, the antibody is suitable for RPPA studies. Figure 4 summarizes the steps for antibody validation.

To facilitate antibody validation the RPPA community is making an international effort to create an upcoming website with detailed information of antibody validation protocols and already validated antibodies [10]. Already available antibodies cover a broad range of pathways, including proliferation, apoptosis, angiogenesis, and epithelial-mesenchymal-transition. An antibody list for RPPA studies can be found for example at the MD Anderson RPPA website [60]. The number of specific and high affinity antibodies targeting the epitope of interest is still limited. Especially the number of phosphor‑specific and other post-translational modification specific antibodies is low [4]. 

### 3.3. Spotting

**Figure 5:**
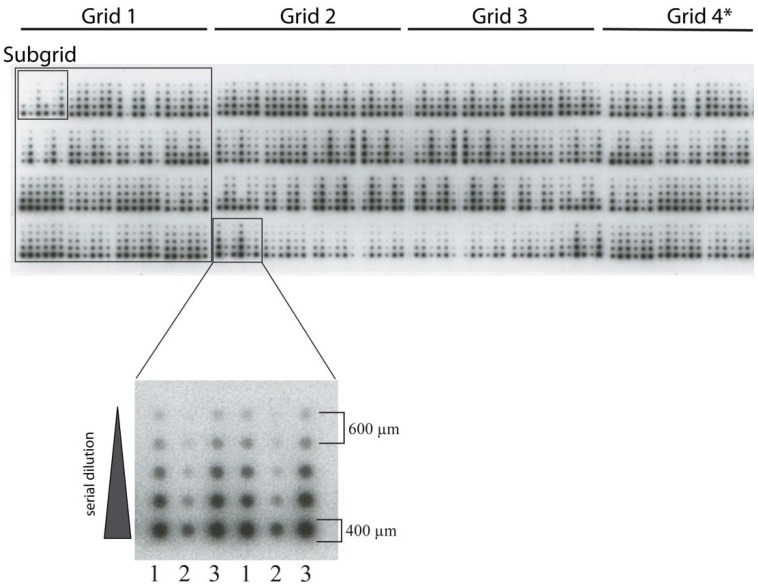
RPPA-spotting pattern detected by chemiluminescent detection. Subgrids comprise 6 samples, each in a five-spot serial dilution plus protein extraction buffer as negative control (36 spots). Grids comprise 16 subgrids and 576 spots (Grid 1–3)/12 subgrids and 432 spots (Grid 4). In total, 180 samples can be spotted per slide (2160 spots). Numbers (1–3) indicate three samples spotted in duplicates. * Smaller grid (3/4 size of Grids 1–3).

The nitrocellulose-coated slides we are using have a size of 7 cm × 2 cm. For RPPA studies only minimal amounts of protein lysates (1 nL of a 2 ng/nL solution) are needed. The samples in our setting are spotted in 3.75 grids (Figure 5). Each complete grid (grids #1–3 in Figure 5) is comprised of 16 subgrids. The smaller grid (grid #4 in Figure 5) is comprised of 12 subgrids. One subgrid consists of three samples in duplicates, most commonly spotted in a five spot two-fold dilution series in duplicates. The dilution series is either performed manually or using an automated liquid handling system (e.g., epMotion from Eppendorf, Hamburg, Germany) in 384-well plates before the spotting process. The dilution series also contains a negative control consisting of protein extraction buffer. On one slide 180 samples can be spotted, including positive and negative controls. For each sample 12 spots (5 fold dilution series, negative control, all in duplicates) are generated. Thus, in total, 2160 spots (12 × 180) are spotted on each slide. Usually proteins are immobilized on nitrocellulose-coated glass using solid pin‑based contact printing, although other printing technologies (piezoelectric or and inkjet spotting) and substrates (e.g., macroporous silicon) have been described [10,61]. Beyond that, the Zeptosens RPPA platform that is based on Planar Wave Guide (PWG) technology permits highly sensitive quantitative protein profiling. Automated systems that are commonly used for RPPA spotting are ArrayIt SpotBot Extreme Microarray Spotter [62] (Arrayit, Sunnyvale, CA, USA), Aushon BioSystems 2470 Microarrayer [19] (Billerica, MA, USA), or SpotArray Microarray printing system [63] (Perkin Elmer, Waltham, MA, USA). Additionally, control samples should also be included in at least duplicates to allow optimal readout and quality control. The spotting devise and other factors, such as temperature and humidity, can affect the spot size. Therefore temperature and humidity should be controlled during the whole spotting process. In our setting a humidity of 60%–65% and a temperature of 14–15 °C to avoid precipitation of SDS, limiting sample evaporation, and optimal sample zone diameter works very well. Other spotting devices or extraction buffer compositions may require different conditions. 

### 3.4. Signal Detection

The immobilized proteins are detected with antibodies whose specificity for the antigen of interest has been validated as described above. The presence of antigen-antibody binding can be detected via near-infrared dyes, chromogenic reporter, chemiluminscence, or planar waveguide technologies [10]. The generated signal is proportional to the primary and, indirectly, to the secondary antibody bound to the spotted proteins, and may be quantified to estimate relative protein concentration. Near-infrared fluorescence dyes are often used [64]. Advantage of fluorescence detection is its large dynamic range; however, photo bleaching and quenching might cause false decreases in the total signal. Chromogenic detection is possible with a multitude of colorless chemical substrates that form a colored product when substrate and enzyme are interacting [65]. This signal detection method is compatible with the use of automated staining stations often used for immunohistochemistry, such as the DAKO autostainer or similar devices, allowing high-throughput staining of RPPA slides. Protein detection by horseradish peroxidase (HRP) that produced light when acting on chemiluminescent substrates (e.g., ECL solution) is shown in Figure 5. In the case of Zeptosens RPPA platform specific analysis software is needed, which is provided by the supplier. For the mentioned RPPA detection methods, it could be shown that it is possible to detect proteins in the fg/mL range with linearity in the pg/mL range [66]. In our hands, chemiluminescent signal detection was the most sensitive and flexible method, allowing even the detection of very low abundant proteins, *i.e.*, transcription factors. However, the following points need to be kept in mind when using chemiluminescence: Although signal detection is very flexible, it is time dependent and, therefore, the comparison of signal intensities from different studies can be a challenge. Bridging samples are one solution to solve this problem. Furthermore, the resolution of the images may be suboptimal which may compromise the quality of the results.

### 3.5. Data Analysis

So far, MicroVigene array software (version 5.6, VigeneTech Inc., Carlisle, MA, USA) is the only software—at least to our knowledge—that was initially designed for RPPA data analysis that includes automatic spot finding and background subtraction. However, beside MicroVigene other programs are also appropriate for RPPA data analysis. These include Array Pro (version 6.3, Media Cybernetics, Rockville, MD, USA), GenePix Pro (version 7.2.29, Molecular Devices, Sunnyvale, CA, USA), and Mapix (version 7.3.1, Innopsys, Carbonne, France) [10]. The spotting of dilution series allows quantification of the protein levels of each sample. To obtain the exact protein concentration, values representing the dilution series should be in a linear range. Positive and negative controls are recommended for use in certification of the measured spot signal. Problems that might occur during data analysis are variations of the background intensities, emerging due to non-specific binding of the secondary antibody or uneven exposure of different parts of a RPPA slide to the reagents used in protein detection (e.g., ECL solution). A recently published study addresses this problem of spatial heterogeneity by the use of positive controls in duplicates. Depending on their spot intensity spatial variation could be corrected for each slide [67]. In addition, Neeley *et al.* propose a normalization step which mainly removes inter-array variability [68]. Another possibility is the spotting of a cell line panel on each slide to be able to calculate spot sizes of the protein of interest normalized to the average spot intensity of the cell lines. In a next step, the calculated data have to be normalized in order to correct potential sources of variability that do not reflect biological differences in protein levels between the investigated samples, such as variations of total amount of protein extracts that were spotted. The usual practice of normalization to a housekeeping (endogenous reference) protein can be problematic due to the limitations of “true” housekeeping proteins. A common method to normalize RPPA data are dye binding methods, *i.e.*, measurements of total protein on one slide per print run with dyes like Fast Green FCF, Sypro Ruby, or colloidal Gold. The signals obtained reflect the total amount of protein per spot immobilized on the solid phase [69,70]. An alternative approach has been shown in a study where an antibody against single-stranded DNA (ssDNA) can be used as a suitable RPPA normalization parameter [71]. 

## 4. Conclusions 

RPPA is a high-throughput method due to the simultaneous analysis of different biomarkers in one sample. One great advantage is that RPPA works with very small amounts of proteins. For this reason, even biopsies are sufficient for RPPA analysis. The implementation of RPPA into clinical practice would help to provide optimal tumor analysis prior and during treatment, enabling to apply the best (*i.e.*, individualized) therapy for each patient, even in the neoadjuvant setting. Due to automated systems that print the proteins of tumor samples on nitrocellulose-coated glass slides, RPPA analysis is simple and rapid. In our opinion, even if the method is limited to high-specific validated antibodies (like for all antibody-based methods), we think that patients, e.g., tumor patients, would benefit from robust tumor marker analysis by RPPA in clinical routine. However, inter-assay reproducibility has to be ensured and clinically validated cut-off levels need to be determined for each tumor marker. Therefore, clinical trials and RPPA-based treatment decisions performed with input material of the highest possible quality will be necessary in the next years.

## 5. Outlook 

RPPA has been developed for quantitative protein measurements of protein levels and to identify post-translationally modified forms in signaling pathways suitable for large scale multiplexed studies. Recent and ongoing research highlights the potential of RPPA for personalized medicine. The information provided by RPPA is already being used in clinical studies to characterize tumors and to select the optimal treatment for each patient. For routine clinical use in the future there are at least two major challenges: (i) The limitation of available, validated and highly specific antibodies [24] and (ii) the poor quality of starting material that can affect the results of the molecular assay [16]. Furthermore, selection of a tumor sample for RPPA and other molecular analysis shall be performed by a pathologist to avoid non-tumorous areas or areas with necrosis. As tumor samples may be heterogeneous and composed of different cell types besides tumor cells (e.g., stromal or inflammatory cells) estimation of the tumor cell content by a pathologist is important. For continued exchange of information with regard to RPPA, an annual Global RPPA Workshop is held which started in 2011. To move RPPA forward to the clinic the “RPPA society” was founded in 2014 during the 4^th^ Global RPPA Workshop held in Paris with the idea of proceeding in sharing information about RPPA guidelines, principles, and improving RPPA technologies. To solve the problem of antibody limitation an antibodypedia-website was already set up which is an open-access resource where already evaluated antibodies are listed that can be used to detect proteins of the human proteome (http://www.antibodypedia.com). However, still, highly specific antibodies against most proteins are missing. Consequently, although new protein biomarkers are continuously identified, their quantitative analysis remains a challenge. 
